# The variegated *canalized-1* tomato mutant is linked to photosystem assembly

**DOI:** 10.1016/j.csbj.2024.10.028

**Published:** 2024-10-24

**Authors:** Micha Wijesingha Ahchige, Josef Fisher, Ewelina Sokolowska, Rafe Lyall, Nicola Illing, Aleksandra Skirycz, Dani Zamir, Saleh Alseekh, Alisdair R. Fernie

**Affiliations:** a Root Biology and Symbiosis, Max Planck Institute of Molecular Plant Physiology, Am Mühlenberg 1, 14476 Potsdam-Golm, Germany; b Plant Sciences and Genetics in Agriculture, The Robert H. Smith Institute of Plant Sciences and Genetics in Agriculture, The Hebrew University of Jerusalem, Herzl 229, 7610001 Rehovot, Israel; c Crop Quantitative Genetics, Center of Plant Systems Biology and Biotechnology, Ruski Blvd. 139, 4000 Plovdiv, Bulgaria; d Department of Molecular and Cell Biology, University of Cape Town, Rondebosch, 7701 South Africa

**Keywords:** Canalization, Variegation, Photosynthesis, Photosystem assembly, Multi-omics, Tomato

## Abstract

The recently described *canal-1* tomato mutant, which has a variegated leaf phenotype, has been shown to affect canalization of yield. The corresponding protein is orthologous to AtSCO2 -SNOWY COTYLEDON 2, which has suggested roles in thylakoid biogenesis. Here we characterize the *canal-1* mutant through a multi-omics approach, by comparing mutant to wild-type tissues. While white *canal-1* leaves are devoid of chlorophyll, green leaves of the mutant appear wild-type-like, despite an impaired protein function. Transcriptomic data suggest that green mutant leaves compensate for this impaired protein function by upregulation of transcription of photosystem assembly and photosystem component genes, thereby allowing adequate photosystem establishment, which is reflected in their wild-type-like proteome. White *canal-1* leaves, however, likely fail to reach a certain threshold enabling this overcompensation, and plastids get trapped in an undeveloped state, while additionally suffering from high light stress, indicated by the overexpression of ELIP homolog genes. The metabolic profile of white and to a lesser degree also green tissues revealed upregulation of amino acid levels, that was at least partially mediated by transcriptional and proteomic upregulation. These combined changes are indicative of a stress response and suggest that white tissues behave as carbon sinks. In summary, our work demonstrates the relevance of the SCO2 protein in both photosystem assembly and as a consequence in the canalization of yield.

## Highlights

• Green and white leaves of *canal-1* mutant show differences on all omics levels.
• green leaves show transcriptional upregulation of photosystem assembly genes.
• white leaves show downregulation of photosystem genes and proteins.
• white leaves are largely defective in photosynthesis and act as carbon sinks.
• SCO2 likely plays an important role as a photosystem assembly factor.

## Graphical Abstract

**Figure:**
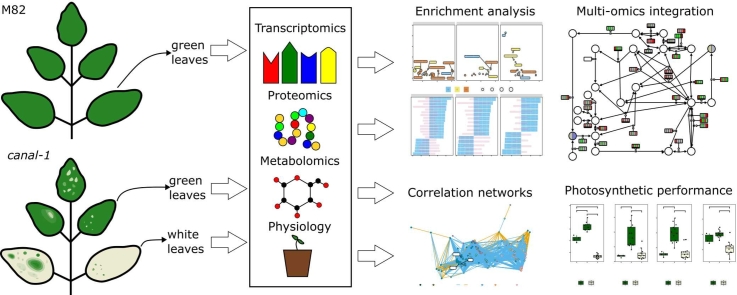

## 1 Introduction

Crop yield remains one of the major focal points in plant breeding being necessary to feed a growing population. Additionally, in the face of increasing environmental variability, it seems necessary to utilize plant breeding to decrease the variability of crop performance. Many studies have investigated mean yield levels in different crop species and found genetic regions or individual genes that affect crop yield. That said the stability of yield has also been long understood to be desirable with this being the focus of studies in barley as early as 1963 [2]. Such studies were extended to include oats and maize [3] and more recently a wide variety of further species including tomato, eggplant, pepper, melon, watermelon, and sunflower [4]. While tomato is not one of the main crops responsible for providing the majority of calories for human consumption, it does play an important role in many diets. It is, furthermore, regarded as a model plant for other fruit-bearing crops [5], as well as being an excellent genetic model system for which a wide range of genomic and post-genomic resources are available [3], [6], [7], [8], [9]. By utilization of different mapping populations, many quantitative trait loci have also been identified in tomato that are responsible for fruit yield [10], [11], [12], [13], [14], [15]. However, in tomato or other crops, much less is known about the genetic factors influencing the variation of yield from plant to plant. A concept to describe the lack of this sort of variation, canalization, was coined by Waddington in 1942 [16]. This has mainly been used in a developmental context, but the concept has recently been applied to also describe quantitative traits [17], [18], [19], [20], [21], [22]. For the purposes of this study, we define canalization as the stability of a phenotype across genetically identical plants.

A recent study of the Zamir lab, using archived mapping data, found a bimodal distribution of stable and variable traits [4]. This study further confirmed this tendency of more and less variable traits, in a common crop garden experiment with many different varieties of crop plants. Further studies in tomatoes have found loci exhibiting both positive and negative effects on yield stability [23]. They also identified a yield canalization mutant with variegated leaves, named *canal-1*. Field-grown plants can vary largely in plant size and accordingly show a strong variation in fruit yield. Fine mapping pinpointed the gene to Solyc01g108200, which is an orthologous gene to SNOWY COTYLEDON 2 (SCO2/AT3G19220) in *Arabidopsis thaliana*. As the name suggests, *A. thaliana* mutants of this gene have snowy white cotyledons but develop regular green leaves under normal conditions [24], [25]. Indeed, the *snowy cotyledon* mutants came out of a genetic screen that was aimed at identifying genes required for proper chloroplast development, particularly in cotyledons. The screen specifically selected mutants with pale cotyledons but normal green true leaves. As yet *sco1*, *sco2,* and *sco3* have been identified and characterized. *Sco1* was identified in a gene encoding the plastid elongation factor G [26]. *Sco2* was identified as a chloroplastic DNA-J-like zinc finger protein that was essential for early seedling survival in Arabidopsis [24], as well as playing a role in leaf variegation in both Arabidopsis [24] and *Lotus japonicus* [27]. By contrast, although *Sco3* could be partially complemented by phytochrome B overexpression the function of the protein remains unknown and intriguingly some mutant alleles also affect the color of the adult leaves [28]. The lack of functional annotation of the protein aside, it is clearly important in normal chloroplast and embryonic development as well as in flowering time and rosette formation [28]. By contrast to the unknown function of SCO3, SCO2 is a protein disulfide isomerase and has been shown to play a role in chloroplast biogenesis [29]. More specifically it is involved in thylakoid development and interacts with other photosystem complex proteins [27]. *SCO2* shows co-expression with genes of the tetrapyrrole biosynthesis pathway but an interaction of the protein to the tetrapyrrole biosynthesis protein HEMA1 could not be shown [29]. Experiments to test whether the protein, interacts with itself, to form a homomer have yielded opposing results [29], [30]. The protein has a conserved zinc finger domain, resembling that of *E. coli* DnaJ, which itself is involved in many processes, related to the transport and folding of proteins as well as the degradation of misfolded proteins [25]. The SCO2 zinc finger domain contains four cysteines that have been shown to be relevant for the catalytic activity of the isomerase activity, for which it likely uses glutathione as an electron donor [30].

As mentioned above in *A. thaliana* mutants of the gene primarily show a cotyledon phenotype but under short-day conditions, true leaves also appear slightly paler [27]. Mutants in *Lotus japonicus* show a variegation phenotype, that resembles the one in tomato [27]. This interesting phenotype led us to characterize the *canal-1* tomato mutant at the molecular level. In the current study, we employ a multi-omics approach encompassing transcriptomics, proteomics, and metabolomics as well as measuring a range of photosynthetic parameters to shed light on the mechanism of yield canalization exhibited by *canal*-1.

## 2 Materials and methods

### 2.1 Cloning and transformation

The gateway cloning system was used to create vectors expressing SCO2 fused C-terminally to eGFP under the 35S-promoter. To achieve this, cDNA was generated from tomato leaf tissue with the maxima first strand cDNA synthesis kit (ThermoFisher Scientific, Waltham MA USA) and used as template for a two-step PCR using Phusion DNA Polymerase (ThermoFisher Scientific, Waltham MA USA) to add adapter sequences to the PCR product. This PCR product was then used in a BP reaction with pDONR221 and then in an LR reaction with pK7FWG2 to create the final construct. *Agrobacterium tumefaciens* GV2260 was transformed with the final construct via electroporation. Tomato plants of the cultivar MoneyMaker were generated via agrobacterium-mediated plant transformation following published protocols [31]. Successful transformation of putative transformants was confirmed by PCR amplification of a portion of the transformed construct, integrated into the plant genome. PCR positive, agrobacterium-free, T0 plants were transferred to the greenhouse for propagation.

### 2.2 Plant material and growth conditions

The *canal-1* mutant was described previously and seeds were supplied by the Zamir Lab [23]. The *ghost-2* mutant, together with the Sioux wild-type plant was ordered from the TGRC. Plants overexpressing the SCO2 protein were generated as described above and used in the generation of T2 plants for experiments. Plants were grown in multiple seasons in different locations. The first growth season was 2017 in a greenhouse in Ashkelon, Israel on a so-called lysimeter assay, which is a high-throughput screening system that monitors water relations for each plant [32]. Seeds of *canal-1* plants and M82 plants were sown at Histill nursery, Ashkelon on September 3rd 2017 and supplied with organic treatment, devoid of dwarfing hormones. The experiment started October 3rd 2017 and tissue samples were collected on November 6th 2017.

Consecutive experiments were conducted at the MPIMP in Potsdam-Golm, Germany and plants were grown as described previously [21]. Plants were grown in 2018 and 2019 in a greenhouse. The experiment including plants overexpressing the SCO2 protein was conducted in 2021. Seeds were directly sown to soil, kept in a growth chamber (York International/Johnson Controls; Cork Ireland), in a (16 h/8 h)-(22 °C/18 °C)-(70 % RH/70 % RH)-(day/night)-cycle, with 150 µmol m^-2^ s^-1^ of additional light. Plants were transferred to individual pots after cotyledons were fully expanded. After four weeks, plants were potted into 18 cm diameter pots and transferred to a greenhouse chamber. Here plants were grown in a (16 h/8 h)-(22 °C/20 °C)-(50 % RH/50 % RH)-(day/night)-cycle and automatically irrigated several times a day by a drip irrigation system. A subset of the plants grown in 2019 received a drought treatment, comprising 50 % of the optimal irrigation. Since plants are irrigated by turning on the valves of the irrigation system, which dispenses water at a steady flow rate, irrigation lines for drought treatment plants were turned on half the respective time. Irrigation rates were adapted according to the growth of wild type plants under optimal conditions and scaled accordingly for plants under drought treatment. Sodium discharge lamps supplied additional light in order to compensate for seasonal differences in light intensity. Plants were fertilized once after potting, onset of flowering and onset of fruit ripening.

### 2.3 Phenotyping

During the growth period, plants were checked three times a week in order to score for the first flower and the first red ripe fruit. For any plants that had not produced fruit at the end of the experiment, we assigned the date of the next regular fruit checking as the date for red ripe fruit. Fruits were harvested when around 80 % of fruits were ripe. At the harvest date, the number of all ripe and unripe fruits per plant was counted and the total fruit weight determined on a scale (Sartorius, Göttingen, Germany). Average fruit weight was determined by dividing the total fruit weight per plant by the total fruit number per plant.

### 2.4 Sampling

Mature non-senescing leaves were sampled and directly snap-frozen in liquid nitrogen. For leaves from *canal-1* mutant plants, whole leaves, leaflets or leaf-parts were selected, which were either almost exclusively pale white or green, trying to minimize highly variegated leaf area. For fruit samples, a section of the pericarp was cut out of a whole red ripe tomato fruit. The cuticle of the fruit was removed and the sample was directly snap frozen in liquid nitrogen. All samples were stored at −80 °C until further processing.

For transcriptomics, proteomics and a subset of metabolomics the same 15 tissue samples, from the 2017 experiment, were used as shown below (Table 1).

**Table 1:** Sample table.

#	Plant ID	Tissue	Genotype	Sample name
1	B11d	white leaves	*canal−1*	Muwl1
2	B14d	white leaves	*canal−1*	Muwl2
3	B11c	white leaves	*canal−1*	Muwl3
4	B11d	green leaves	*canal−1*	Mugl1
5	B14a	green leaves	*canal−1*	Mugl2
6	B11c	green leaves	*canal−1*	Mugl3
7	B17c	green leaves	M82	Wtgl1
8	B17a	green leaves	M82	Wtgl2
9	I06c	green leaves	M82	Wtgl3
10	B14a	stem	*canal−1*	Mus1
11	B14d	stem	*canal−1*	Mus2
12	B12d	stem	*canal−1*	Mus3
13	I05b	stem	M82	Wts1
14	B05b	stem	M82	Wts2
15	B06c	stem	M82	Wts3

### 2.5 RNA extraction

Total RNA was extracted according to a published lithium chloride precipitation protocol [33]. A NanoDrop_TM_ One spectrophotometer (ThermoFisher Scientific, Waltham, MA, U.S.A.) was used to estimate RNA concentration. For the digestion of residual DNA, 10 µg of raw RNA was digested with the Invitrogen Turbo DNAse free kit (Invitrogen/Thermo Fisher Scientific, Waltham, MA, U.S.A.). The Bioanalyzer 2100 (Agilent; Santa Clara, CA, U.S.A.) was used to determine RNA quality and quantity.

### 2.6 Orthology

For orthology search, OrthoFinder v2.5.2 (Emms and Kelly, 2019) was utilized, using the diamond_ultra_sens algorithm with proteomes using only the primary transcript of *Arabidopsis thaliana* Araport11, tomato (*Solanum lycopersicum*) ITAG4.0, potato (*Solanum tuberosum* v6.1), *Lotus japonicus* Lj1.0v1, maize (*Zea mays* RefGen_V4) and rice (*Oryza sativa* v7.0) obtained from Phytozome [34], [35], [36], [37], [38], [39]. Orthologs of photosynthetic genes were found by matching genes from *Arabidopsis thaliana* found in literature [40], [41], [42], [43], [44], [45] to the discovered orthogroups. A resolved gene tree of the glutaredoxin orthogroup was constructed with ggtree [46].

### 2.7 Protein visualization

Putative protein structure was obtained from AlphaFold [47], [48] and visualized with Mol* Viewer [49].

### 2.8 RNAseq analysis

Library preparation and RNA sequencing was performed by the MPIPZ Cologne. Libraries were prepared by a polyA enrichment. Reads were acquired by a HiSeq3000 sequencer (Illumina, CA, U.S.A.) as 2 × 150 bp paired-end reads, with a sequencing depth of 20,000,000 reads. Raw reads were mapped to the nuclear and organellar genome via LSTrAP [50]. Differential gene expression was performed with deseq2. Genes with an FDR-adjusted p-value ≤ 0.1 and a log2-fold-change ≥ 1 were considered differentially expressed based on recommendations in the literature [51], [52]. The expression matrix as produced by LSTrAP and a deseq2-normalized expression matrix together with log2-fold changes and adjusted p-values of the respective contrasts can be found with the supporting information (Supplementary Data S 3 + Supplementary Data S 4).

Solgenomics Gene ID to Entrez Gene ID correspondence was inferred by aligning the ITAG4.0 proteome against the NCBI Refseq SL3.1 proteome with Diamond v.0.9.9. Alignments were filtered for 100 % identity and at least 95 % query cover as well as being on the same chromosome in both assemblies. Solgenomics Gene IDs mapping to more than one Entrez Gene ID were ranked based on the absolute distance of their start regions and only the entry with the smallest distance was retained. A correspondence table can be found with the supporting information (5).

Gene ontology enrichment analysis and gene set enrichment analysis (GSEA) was performed with the help of the packages topGO and fgsea. Bubble plots were inspired by the GOplot package [53]. Accordingly the z-score was calculated as detailed below (Formula 1).

$$z-\mathrm{score}=\;\frac{\left(\mathrm{up}-\mathrm{down}\right)}{\sqrt{\left(\mathrm{count}\right)}}$$

Formula 1: Z-score calculation for bubble plots. up: number of upregulated genes, down: number of downregulated genes, count: total number of genes.

### 2.9 Metabolite extraction

Plant tissue was ground to fine powder with a mixing mill (Retsch; Haan, Germany). Here, plant tissue was added to a pre-cooled stainless steel grinding jar loaded with a 20 mm steel ball and milled for 1 min at 30 Hz. Aliquots of 50 mg plant tissue powder were weighed with a fine scale and used for combined extraction of metabolites, proteins and pigments, which was carried out exactly according to a previously described protocol [54].

### 2.10 Pigment determination

The aliquot of 100 µl taken from the lipophilic phase of the MTBE extraction was diluted 10-fold with methanol and the extracts were measured under an Epoch2 96-well plate reader (Biotek/Agilent; Santa Clara, CA, U.S.A.). Spectrum was measured at 470 nm, 652 nm and 665 nm. Assuming a pure methanolic extract, pigment concentration was calculated with a pathlength correction of 0.51 as described previously [55], [56].

### 2.11 Proteomics

Proteins were purified from the pellet of the MTBE extraction according to previously published protocols [54], [57]. Here, the protein pellet was dissolved in 50 µl denaturation buffer (50 mM Ammonium bicarbonate pH 8 (Ambic), 2 M Thiourea, 6 M Urea) and sonicated in a sonication bath for 15 min. After determining protein concentration by Bradford Assay (Bio-rad, Hercules, CA, USA), a volume containing 50 µg of protein was added to a new tube and filled to 20 µl with denaturation buffer. After adding 2 µl of reduction buffer (5 mM DTT) samples were incubated for 30 min at room temperature. Samples were then incubated for another 30 min at room temperature before adding 2 µl alkylation buffer (30 mM iodoacetamide). To the samples 5 µl LysC/Trypsin mix (50 mM AmBic; 20 µg protein) was added and an additional 15 µl AmBic buffer (50 mM Ambic pH 8) was added to achieve a final urea concentration of 4 M. Samples were then incubated for 2 h at 37 °C. After adding 120 µl Ambic buffer to achieve a urea concentration of 1 M, samples were incubated at 37 °C overnight. After that, samples were acidified with 1.65 µl Trifluoroacetic acid (TFA) to a final TFA concentration of 1 % (v/v).

For protein desalting, Sep-Pak filter columns were placed on top of a vacuum manifold, washed first with 1 ml MeOH, then with 1 ml Solution B (0.1 % TFA (v/v), 80 % acetonitrile (v/v)) and then two times with 1 ml Solution A (0.1 % TFA (v/v)), making sure to apply vacuum until all liquid passed through the column between each wash. Samples were then loaded onto columns and vacuum was applied until all liquid had passed through the column. Columns were again washed two times with 1 ml Solution A, letting all liquid pass through the column, before placing 2 ml tubes under the outlets. Peptides were eluted with 600 µl Solution C (0.1 % TFA (v/v), 60 % acetonitrile (v/v)) and the flow through was collected. Peptides were transferred to 1.5 ml tubes and dried in the Speed vacuum concentrator for 3 h and stored at −20 °C until further use.

Proteomics mass spectrometry was performed as described before [58]. Isolated peptides were resuspended with MS loading buffer and run on an ACQUITY UPLC M-Class System (Waters, Wilmslow UK) coupled to a Q Exactive HF Orbitrap mass spectrometer (Thermo Fisher Scientific, Waltham MA, USA).

Proteomics data were analyzed with the MaxQuant software [59] and the tidyproteomics package [60]. Missing values were imputed by the half minimum per protein and differentially expressed proteins were estimated with two-sided t-tests after normalization with limma [61]. Proteins were considered differentially expressed at a log2-fold-change ≥ 1 and an FDR-adjusted p-value ≤ 0.1. Protein groups, that contained multiple proteins were split into all individual proteins. The raw data as produced by the MaxQuant software as well as a table showing raw intensities together with log2-foldchanges and p-values can be found in the Supporting information (Supplementary Data S6 + Supplementary Data S7).

### 2.12 Metabolomics

Primary metabolites were measured via gas chromatography-mass spectrometry (GC-MS) according to previously used protocols [62]. Secondary and lipophilic metabolites were measured via polar liquid chromatography-mass spectrometry (LC-MS) exactly according to the described protocols [63].

Metabolites were annotated according to in-house libraries, the Golm Metabolome Database (GMD, http://gmd.mpimp-golm.mpg.de/) and literature [64], [65], [66]. Heatmaps were created with the ComplexHeatmap package [67]. Metabolic pathway enrichment was performed with the web-version of MetaboAnalyst 5.0 (https://www.metaboanalyst.ca/MetaboAnalyst/home.xhtml) [68].

Metabolite values from samples extracted from the same source material in 2017 and 2021 were averaged, after normalization to wild type samples, as they constitute technical replicates. All metabolite data are reported following recently updated metabolite reporting standards Supplementary Data S 1 [69], [70].

### 2.13 Photosynthesis measurements

Photosynthetic parameters were estimated in two independent experiments. The first measurement was done on control plants in the experiment from 2019, using a LI-6400–40 fluorometer (LI-COR Biosciences GmbH, Bad Homburg, Germany). Measurements were done on light-adapted leaves under 400 µE m^-2^ s^-1^ PAR, with 10 % blue light, 400 ppm CO_2_, leaf temperature of 25 °C and a flow of 300 µmol s^-1^ as described before [71]. The second measurement was done on plants in the experiment from 2021 including overexpressing plants, using a handheld MultispeQ device V2.0 (Photosynq Inc., East Lansing, MI, U.S.A.)[72]. Measurements were performed under ambient light and CO_2_ concentration, using the Rides 2.0 protocol. To account for fluctuating light intensity throughout the measurement, the time of day and the square root of PAR were added as factors to the anova. Outlier values, which were more than three times the interquartile range below the lower or above the upper quartile were removed before statistical analysis.

### 2.14 Omics integration

Different omics datasets were integrated by the help of Pathview [73] and SBGNview [74]. First Vanted (v2.8.3) [75] and the SBGN-ED plugin [76] were used to download KEGG pathway maps, which were transformed into SBGN process description maps. KEGG REST API was used for gene and compound mapping (https://www.kegg.jp/kegg/rest/keggapi.html). For the glycine cleavage system (EC 1.4.1.27) we manually combined the ECs 1.4.4.2, 1.8.1.4 and 2.1.2.10 and their corresponding KEGG orthologies. Pathway maps were labelled accordingly, and their layout optimized with the help of Vanted and newt editor [75], [77], [78]. Finally, omics data was mapped to pathway maps with the SBGNview package [74] with minor modifications currently forked to https://github.com/micwij/SBGNview.

### 2.15 Correlation network

Correlation networks were constructed by calculating the Spearman-correlation value for the 15 samples for which all omics-data were available (Table 1). Different thresholds were used for different networks, but to reduce computational burden correlation values below 0.75 were directly filtered out, when gathering data. Correlation networks were generated with igraph and network graphs were created with the help of ggraph and graphlayouts [79], [80], [81].

### 2.16 Computational analysis, used packages, webtools and software

Computational analysis and visualization was performed with R statistical software v4.2.1 in the Rstudio environment or on our in-house high performance computing platform. In addition to previously mentioned packages we also used the following packages for data handling and visualization: tidyverse, ggtext, ggpubr, gggenes, ggrepel, ggbeeswarm, cowplot, openxlsx, broom, car, deseq2, apeglm, topGO, fgsea, clusterprofiler, viridisLite, lme4, broom.mixed, here, extrafont [82], [83], [84], [85], [86], [87], [88], [89], [90], [91], [92], [93], [94], [95], [96], [97], [98], [99]. Benchling [Biology Software] (https://benchling.com) was used for sequence alignments. Serial Cloner v2.6.1 (SerialBasics http://serialbasics.free.fr/Serial_Cloner.html) and Benchling [Biology Software] (https://benchling.com) were used to plan cloning of constructs. Colors were picked from a colorblind-friendly scale as suggested previously [100] with the help of a webtool (https://davidmathlogic.com/colorblind/#%23D81B60-%231E88E5-%23FFC107-%23004D40), which was also used to pick additional colors. The bibtex package was used to export R package citations into.bib files [101].

### 2.17 Statistical analysis

As many experiments had an uneven number of samples a pairwise-wilcox test was performed. Equally distributed samples were analyzed by two-sided t-tests. Unless otherwise stated unadjusted p-values are shown.

### 2.18 Accession Numbers

Sequence data from this article can be found in the solgenomics database. Accession numbers of genes and proteins are listed in the supporting information (Supplementary Data S 3 - Supplementary Data S 7).

## 3 Results

### 3.1 Genetic, phenotypic and physiological characterization of the *canal-1* mutant

**Fig. 1:**
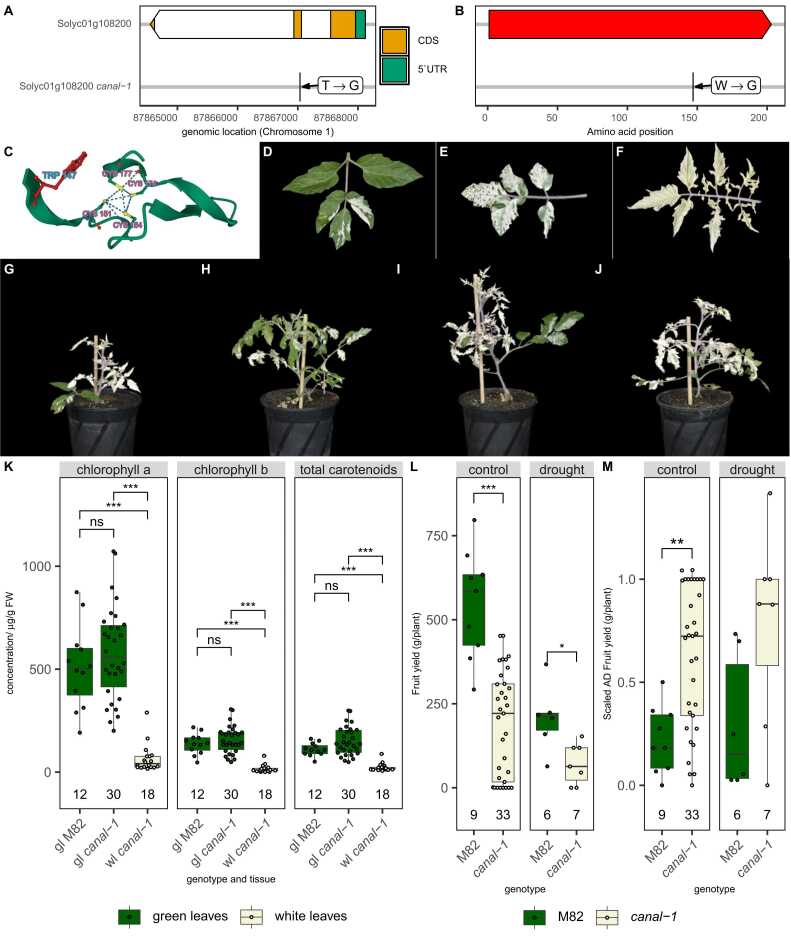
**Genotype and Phenotype of *canal-1* mutant** A, Schematic representation of gene model of Solyc01g108200, with coding sequence (CDS; orange) and 5’ untranslated region (5’UTR, green). Arrowhead of gene model box indicates gene direction. Label shows nucleotide changes in one letter code and arrow points to location of mutation. B, Schematic representation of protein sequence (top red box). Label shows amino acid changes in one letter code and arrow points to location of mutation. C, 3D-model of conserved zinc finger domain of wild type SCO2 protein. Conserved cysteine residues (pink text and yellow side chain sulfur atoms) as well as the tryptophan (blue text and red side chain), that is altered in the *canal-1* mutant are highlighted. D-E, Phenotype of leaves from *canal-1* mutant with varying degrees of variegation. G-J, Habitus of different *canal-1* plants. K, Pigments measured in green and white leaves of mutant and wild type plants. Subpanels show different pigments. L, Fruit yield of M82 and *canal-1* plants. Subpanels show control and drought conditions. M, Scaled absolute deviation (AD) from the median fruit yield calculated from L. Subpanels show control and drought conditions. In G-J, images have been edited to remove parts of the bamboo stick beyond the size of the plant. All boxplots show the interquartile range (IQR) between the first and the third quartile as the box, with the median indicated by a black center line. Whiskers extend from quartiles to most extreme points with a maximum of 1.5 x IQR. Points beyond that range are considered outliers but overlaid by individual data points, shown as quasirandom. Boxes and points are filled according to tissue (green leaves: green, white leaves: beige) in K and according to genotype (M82: green; *canal-1*: beige) in L-M. Number of samples per group is shown above x-axis. Statistical significance was estimated by pairwise-wilcox test for K and kruskal-wallis test for L-M and significant differences are indicated by a bracket and asterisks (*: p ≤ 0.05; **: p ≤ 0.005; ***: p ≤ 0.0005).

The *canal-1* mutant has a missense mutation from T to G, 923 bp downstream of the start codon of Solyc01g108200, leading to a Trp (W) 147 to Gly (G) conversion (Fig. 1 A + B)[23].

We obtained a 3-D model of the protein in tomato from alphafold and visualized the spatial orientation of different key residues (Fig. 1 C). The altered amino acid residue (TRP147; red side chain and blue label) is in close proximity to four conserved cysteines (CYS151, CYS154, CYS174, CYS177; yellow side chain sulfur atoms and pink label), which are putatively responsible for the catalytic activity (Fig. 1 C) [25], [30]. The variegation phenotype of *canal-1* mutant leaves ranges from almost completely green over differently patterned variegated to completely pale white-beige (Fig. 1 D-F). Additionally, the distribution of green and white leaf area varies from plant to plant (Fig. 1 G-J). This is in agreement with what has been observed in the field [23]. We estimated the content of different pigments in wild-type green leaves as well as in mutant leaves, through spectroscopic measurements (Fig. 1 K). As expected, white *canal-1* leaves were almost completely devoid of both chlorophyll a and b and also of carotenoids. Interestingly, there were no statistically significant differences in any of these pigments, when comparing green mutant leaves to wild-type leaves. Stems from *canal-1* plants appeared as intermediate, having a strongly reduced albeit detectable level of chlorophyll (Supplementary Figure S 1). We also measured the fruit yield at maturity of plants grown in the greenhouse under both control and drought conditions (Fig. 1 L). Unsurprisingly, *canal-1* plants produced lower fruit yield than wild-type plants under control and drought conditions. We also wanted to estimate the effect of the mutation on yield stability as seen in the field. However, given that our experiments did not have the same layout as the so-called canalization replication trials performed in the field [23], instead of using the coefficient of variation (CV), we estimated variation by the scaled absolute deviation from the median (Fig. 1 M). Indeed, *canal-1* mutants showed a much higher relative variation of fruit yield when compared to wild-type plants, despite not showing the large variation in plant size that was observed in the field [23]. This effect was, however, only significant under control conditions. However, it is possible that it was masked by the small number of plants that we subjected to drought stress.

### 3.2 Transcriptomics reveal upregulation of photosystem assembly factors in green *canal-1* leaves

**Fig. 2:**
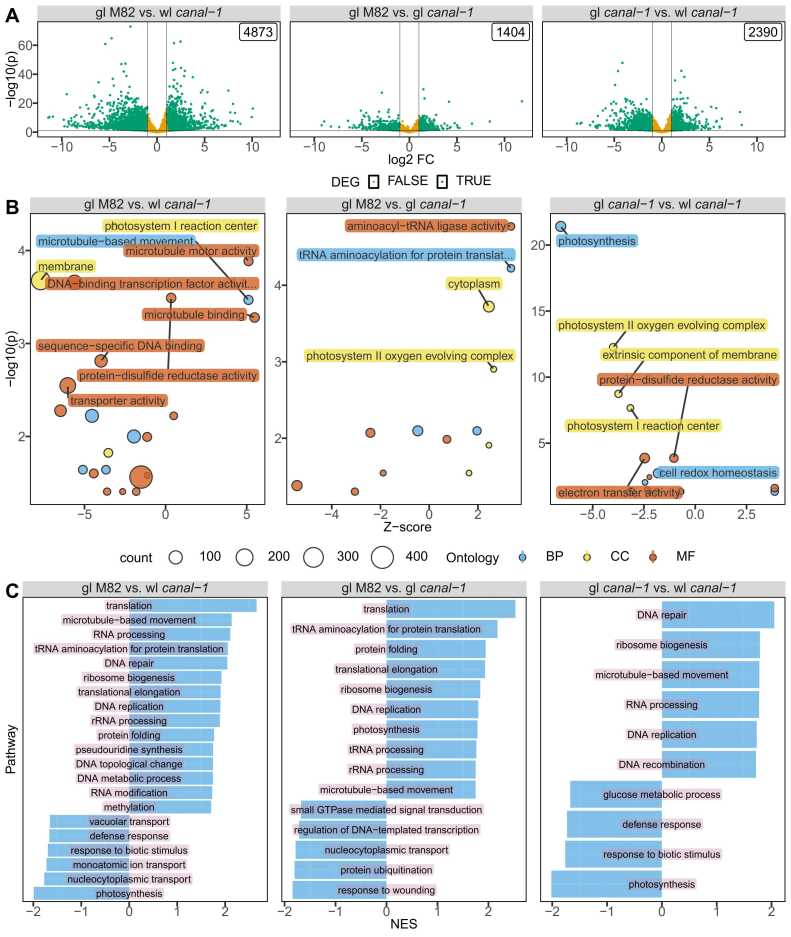
Transcriptome of canal-1 leaves compared to M82 leaves. A, Volcano plot of differentially expressed genes in different contrasts. Differentially expressed genes are shown in green and non-DE genes are shown in yellow. Label in top right-hand corner shows the number of DE genes of the respective contrast. Vertical and horizontal lines are thresholds for differential gene expression for fold-change and p-value respectively. B, Bubble plot with enriched gene ontologies according to topGO. Bubbles and labels are colored according to ontology and sized based on the number of genes in the respective term. C, Barplot of enriched gene sets according to fgsea. gl: green leaves; wl: white leaves; BP: biological process, cc: cellular component, MF: molecular function; NES: normalized enrichment score. (DEG: p ≤ 0.05 & |log2FC= ≥ 1).

Due to the pigmentation phenotype, we considered that leaf tissues would be the most interesting tissue for a detailed characterization. We, therefore, selected three samples from wild-type leaves and stems as well as green and white leaves and stems from *canal-1* plants for RNA sequencing (Table 1). We mapped transcript reads with LSTrAP and analyzed differentially expressed genes (DEGs) via deseq2. The PCA already showed a clear separation of tissues and also highlighted white mutant leaves as being more distinct from green leaves of both mutant and wild type plants (Supplementary Figure S 2). For the analysis, we compared mutant white leaves to both mutant green leaves and wild-type green leaves, as well as green leaves and stems between wild-type and mutant (see Venn diagrams in Supplementary Figure S 3 A + B). Perhaps to be anticipated, we saw the most DEGs when comparing white *canal-1* leaves to green M82 leaves (4873 DEGs), followed by white versus green leaves of the mutant (2390 DEGs, Fig. 2 A).

With 1404 DEGs the comparison between green leaves of *canal-1* and M82 green leaves still showed some substantial differences. However, these were relatively minor as demonstrated by the smaller log2-fold change and –log10(p)-values in the volcano plot (Fig. 2 A).

We next investigated which kinds of genes were changed in the different comparisons by conducting gene ontology (GO) enrichment analysis (Fig. 2B). Since GO enrichment does not consider up- or downregulation of genes within an enriched term, we calculated a z-score by subtracting the number of downregulated genes from the number of upregulated genes and divided this by the square root of the total count of genes in each term (see Formula 1). Accordingly, a positive z-score indicates more upregulated, than downregulated genes and a negative z-score indicates more downregulated than upregulated genes. When comparing white *canal-1* leaves to M82 leaves we found terms such as “photosystem I reaction center” and “protein disulfide oxidoreductase activity” enriched (Fig. 2B left and Supplementary Table S 1). Although photosynthesis as a GO term for a biological process was also enriched here the enrichment was much clearer when comparing white leaves of *canal-1* to green leaves (Fig. 2B right and Supplementary Table S 1 and Supplementary Table S 2). The comparison of green mutant and wild-type leaves yielded similar terms as the other comparisons (Supplementary Table S 3). Interestingly, the term “photosystem II oxygen evolving complex” is slightly upregulated in green leaves of *canal-1* plants compared to M82 leaves (Fig. 2 B, Supplementary Table S 1 and Supplementary Table S 3). Furthermore, terms related to aminoacylation were also enriched (Fig. 2B middle and Supplementary Table S 3). Enriched terms in the comparison of stem samples were rather related to cell wall processes (Supplementary Table S 4).

We also utilized gene set enrichment analysis (GSEA), which not only considers DEGs but takes all genes into consideration and ranks them according to their fold-change and *p*-value (Fig. 2 C). GSEA highlighted translation as the most strongly enriched term in both comparisons of white and green leaves of mutant plants compared to wild type plants, but not when comparing the two different leaf types of the mutant (Supplementary Table S 6 - Supplementary Table S 8). Photosynthesis was the strongest downregulated term in the comparisons of white mutant leaves either to wild type leaves or to green mutant leaves (Fig. 2 C). In agreement with the GO analysis, photosynthesis is upregulated in the green leaves of mutant plants, when compared to wild type plants. Irrespective of their color, mutant leaves showed an upregulation of different DNA-, RNA, and protein-related terms, when compared to M82 leaves. The stem comparison only revealed a small number of enriched terms, related to lignin or protein (Supplementary Figure S 6, Supplementary Table S 9).

Because we cannot exclude that some of the observations from white mutant leaves are pleiotropic effects from incomplete photosystems, rather than their cause we turned our attention again towards the comparison of green leaves of mutant and wild type. One individual gene that is highly upregulated in green *canal-1* leaves but not in white *canal-1* leaves in comparison to wild type leaves is Solyc08g048410 (Supplementary Data S 4), which codes for a protein annotated as Ubiquitin carboxyl-terminal hydrolase 21[102]. Proteins belonging to this type of enzyme are deubiquitilating enzymes, meaning they can remove ubiquitin monomers to restore proteins to their original form [103]. Together with the enriched aminoacylation terms from GO and the enrichment of protein folding in GSEA, this could mean that more proteins are available in green leaves that survive degradation and can be correctly folded to contribute to photosystem assembly.

The discovery of the enriched protein disulfide oxidoreductase GO term (GO:0015035) and the fact, that SCO2 is a protein disulfide isomerase prompted us to investigate genes related to protein disulfide activity. Among genes belonging to the GO:0015035 one group of genes stood out (Supplementary Figure S 5). All of the genes showed, relatively consistently, low or no expression in wild type green leaves, an increased level in *canal-1* green leaves, and even higher expression in white mutant leaves. We noticed that these genes carry consecutive gene IDs and when checking the results from our orthology search, we realized, that they all belong to the same orthogroup, also including the CC-type glutaredoxin (ROXYs), *ROXY16* and *ROXY17* [104], [105](Supplementary Figure S 7). We also investigated the terms photosystem I reaction center and photosystem II oxygen evolving complex (Supplementary Figure S 8 & Supplementary Figure S 9). Genes from the photosystem I reaction center term showed a regular pattern of differential expression between white mutant leaves to both mutant and wild-type green leaves, with a lower abundance in white mutant leaves (Supplementary Figure S 8). For the term photosystem II oxygen evolving center, expression was also mostly significantly lower in white mutant leaves than in green mutant leaves and/or green wild type leaves but for some genes, the expression was significantly higher in green *canal-1* leaves in comparison to green M82 leaves (Supplementary Figure S 9).

**Fig. 3:**
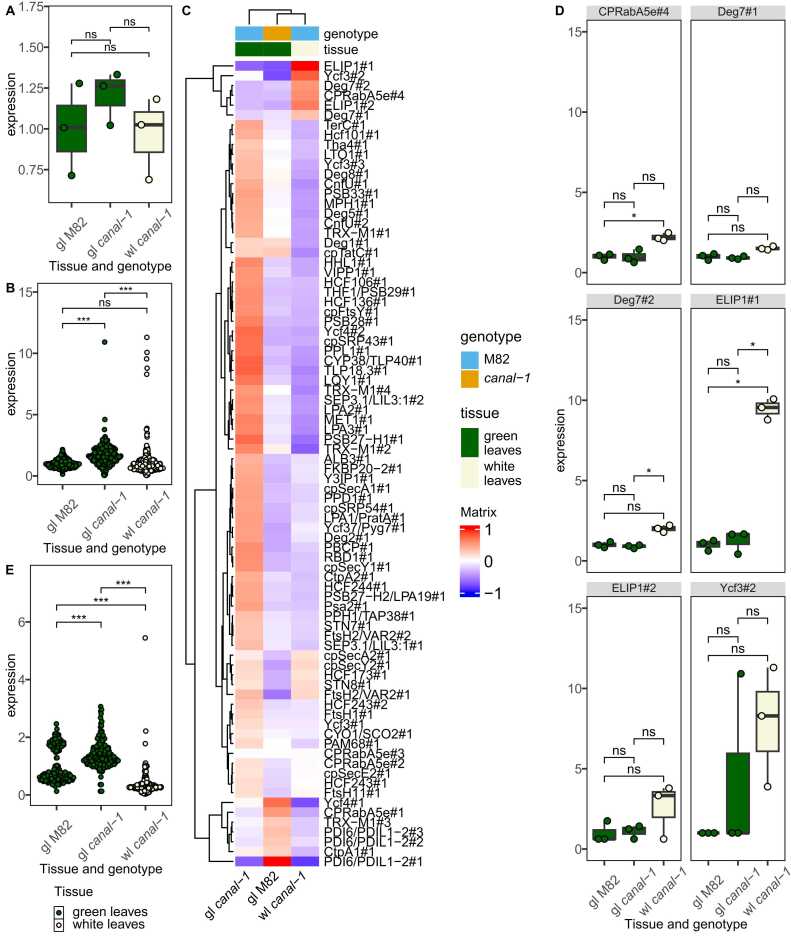
Transcriptome of different photosystem components in *canal-1* and M82 leaves based on deseq2-normalized counts. A, Expression of Solyc01g108200. B, Expression of photosystem assembly components. C, Heatmap of level-scaled expression levels for individual photosystem assembly components with color scale ranging from −1 to 1. D, Expression of orthologs to Deg7, ELIP1, CPRabA5e and Ycf3. E, Expression of protein complex genes of photosystems. Boxes and points are filled according to tissue (green leaves: green, white leaves: beige). To facilitate scaling to wild type samples, zero values were imputed by the half-minimum per gene. Statistical significance was estimated by pairwise-wilcox test for B and E and extracted from differential gene expression analysis for A + D. Significant differences are indicated by a bracket and asterisks for A + D (*: p ≤ 0.05 & |log2FC= ≥ 1) and for B and E (*: p ≤ 0.05; **: p ≤ 0.005; ***: p ≤ 0.0005).

Due to the likely role of the SCO2 protein and the photosynthesis-related terms appearing in the enrichment analysis, we investigated the expression of different genes involved in photosystem assembly more deeply (Fig. 3).

First, as the protein under investigation is itself putatively involved in thylakoid assembly, we checked its expression in mutant and wild-type leaves (Fig. 3 A). The gene, however, did not appear to be differentially expressed in any of the comparisons. To expand our scope to all potential photosystem assembly genes, we curated a list of known photosystem components and photosystem assembly genes in *A. thaliana* from the literature [40], [41], [42], [43], [44], [45] and matched them to the tomato genes via orthology searches (Supplementary Table S 5). When considering all these genes, we could see that green leaves of *canal-1* plants showed a significant upregulation of the expression in comparison to both white *canal-1* leaves and wild-type leaves (Fig. 3 B). White *canal-1* leaves, however, showed a wild-type level of expression of photosystem assembly genes, with the exception of a few outliers that displayed a strong upregulation. We visualized the level-scaled expression of these genes individually in a heatmap and could confirm that the vast majority shows the highest expression in green mutant leaves, with the exception of a small cluster of genes that showed the highest expression in white mutant leaves and another cluster of genes with the highest expression in wild-type leaves (Fig. 3 C). The assembly genes, which are highly expressed in white *canal-1* leaves are orthologs of ELIP1 (Solyc09g082690/700) and DEG7 (Solyc03g043660) as well as CPRabA5e (Solyc11g012460) and Ycf3 (Solyc00g500145), some of which are differentially expressed between white mutant leaves and/or green mutant and wild type leaves (Fig. 3 C + D). We were interested to see how these differences affect the gene expression of the proteins which finally comprise the photosystems. We, therefore, filtered the transcriptome dataset for genes orthologous to known photosystem components in *A. thaliana* (Fig. 3 E). In contrast to the assembly factors, the photosystem components are significantly reduced in the white leaves of the mutant compared to mutant and wild-type green leaves. Additionally, expression of these genes is also significantly increased in green *canal-1* leaves, relative to M82 leaves.

### 3.3 Proteomics reveals a wild type like proteome of green *canal-1* leaves

**Fig. 4:**
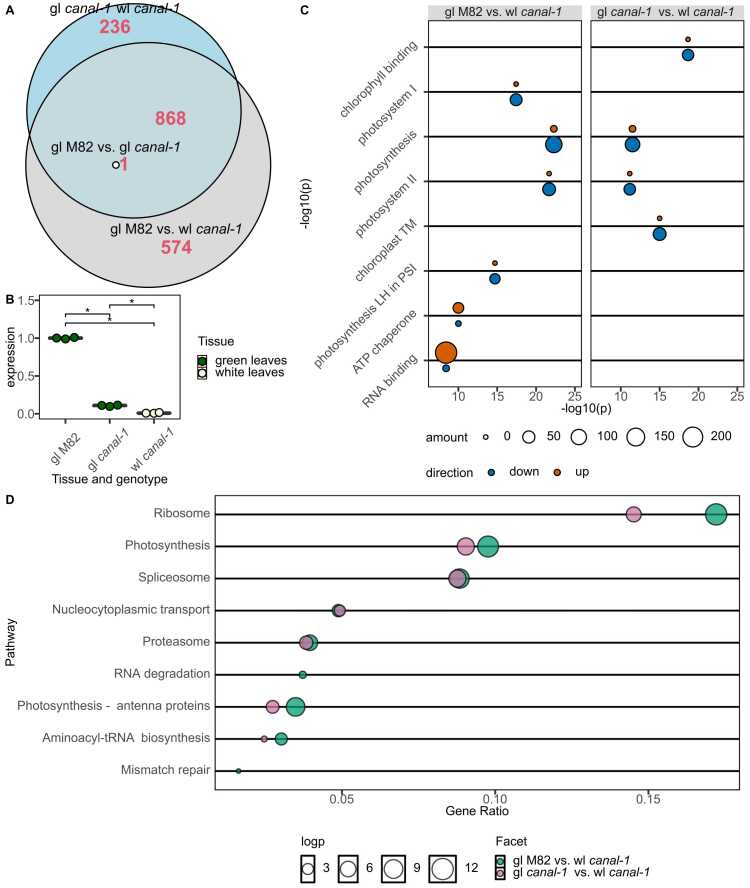
Differentially abundant proteins across different contrast. A, Euler plot of overlapping differentially abundant proteins between different contrasts. B, Relative abundance of oxygen evolving complex in mutant and wild type leaves. C, Bubble plots of top enriched GO terms according to topGO. Bubbles are colored according to up- or downregulation and sized based on the number of genes in the respective term. D, Enriched KEGG terms according to clusterProfiler. gl: green leaves; wl: white leaves. Full GO term names are shown in Supplementary Table S 10.

Having assessed transcriptional changes we next investigated which of these translated to changes at the proteome level, for which we again selected samples from white and green *canal-1* leaves and M82 leaves. When performing a PCA, we could once again see the separation of several groups of samples (Supplementary Figure S 10). Interestingly here, the white mutant leaves clustered even further away from green mutant and wild type leaves as compared to the PCA of RNAseq data (Supplementary Figure S 2). Similarly, as for the transcriptome data, we determined differentially abundant proteins (DAPs) (Fig. 4).

For this purpose, we used an established shotgun proteomics platform and mapped the peptides to the tomato proteome UP000004994. We were able to identify a total of 6315 protein groups in this manner which could be further split, yielding 7729 different proteins. Interestingly, *canal-1* green leaves appeared to be wild-type like at the proteome level (Fig. 4). When comparing the *canal-1* white leaves to *canal-1* and M82 green leaves, we found 1105 and 1443 DAPs, with 869 DAPs overlapping between them (Fig. 4A). In addition to 868 overlapping between these comparisons, there was a single protein A0A3Q7EIF5, which additionally showed differential expression when comparing *canal-1* green leaves to M82 leaves, making it the only protein showing differential abundance between green mutant and wild type leaves (Fig. 4 A). This protein was annotated as a component of the oxygen evolving center (OEC) of photosystem II (Fig. 4B). This is surprising, considering that on a transcriptomic level the term “photosystem II oxygen evolving complex” showed enrichment in the comparison of green mutant and wild type leaves and expression was generally higher in green mutant leaves (Fig. 2, Supplementary Figure S 9). This result is further interesting, under the same assumption made for the transcriptome, that results from white leaves may contain a number of pleiotropic effects, whereas observations from green mutant leaves may be characteristic of direct effects of the mutation. We used the GO terms to perform another enrichment analysis (Fig. 4C). For the comparison of white mutant leaves and green wild type leaves the most significantly enriched term was photosynthesis and for the comparison of white and green mutant leaves it was chlorophyll binding (Fig. 4C). Besides other terms related to photosynthesis, we found also terms related to RNA binding and ATP-dependent protein folding chaperone (Fig. 4C, Supplementary Table S 11 and Supplementary Table S 12).

Having the opportunity to directly translate UniprotIDs into EntrezIDs, we also performed an enrichment analysis using KEGG terms (Fig. 4D). Again, we found photosynthesis terms enriched, but also the terms “Spliceosome”, “Ribosome” and “Proteasome”. (Supplementary Table S 13 + Supplementary Table S 14). In general, the pattern of enriched terms seems relatively congruent between both comparisons, highlighting the common differences between white *canal-1* leaves to both green *canal-1* and M82 leaves (Fig. 4C and D).

**Fig. 5:**
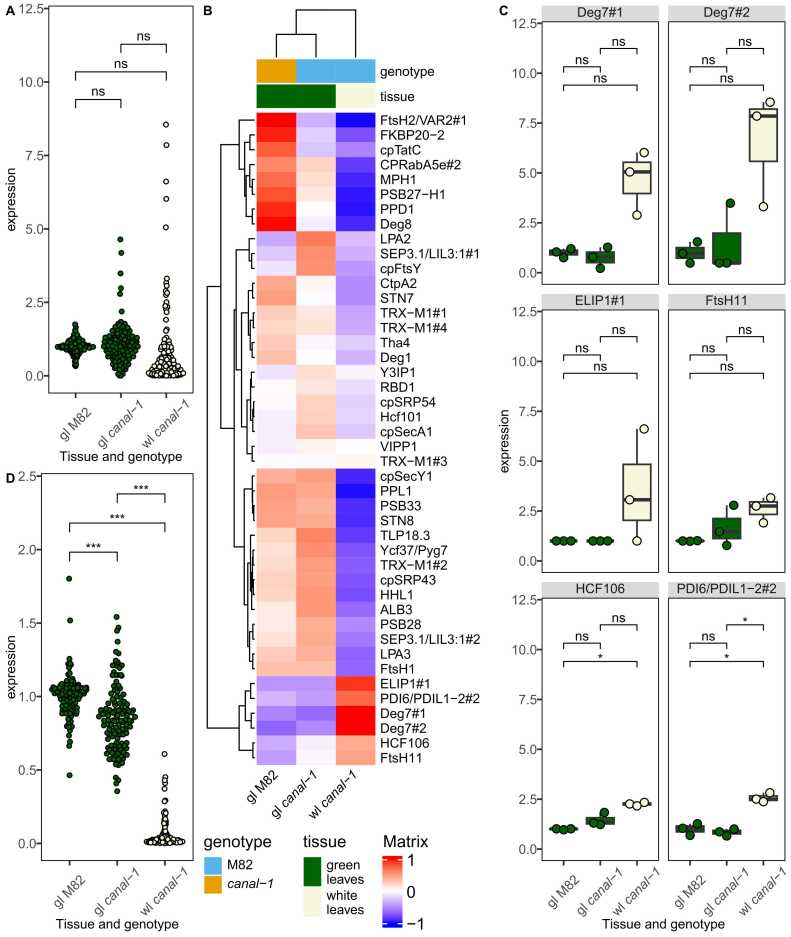
Protein levels of photosystem components and assembly factors in *canal-1* and M82 leaves. A, Assembly factors of photosystem components. B, Heatmap of level-scaled protein levels for individual photosystem assembly components with color scale ranging from −1 to 1. C, Individual proteins with high abundance in white mutant leaves. D, Abundance of photosystem components proteins in leaves. Statistical significance was estimated by pairwise-wilcox test for A and D and extracted from differential gene expression analysis for C. Significant differences are indicated by a bracket and asterisks for C (*: p ≤ 0.05 & |log2FC= ≥ 1) and for A and D (*: p ≤ 0.05; **: p ≤ 0.005; ***: p ≤ 0.0005).

Similar to the transcriptome data, we also took a closer look at protein levels of components and assembly factors of the photosystems (Fig. 5).

**Fig. 6:**
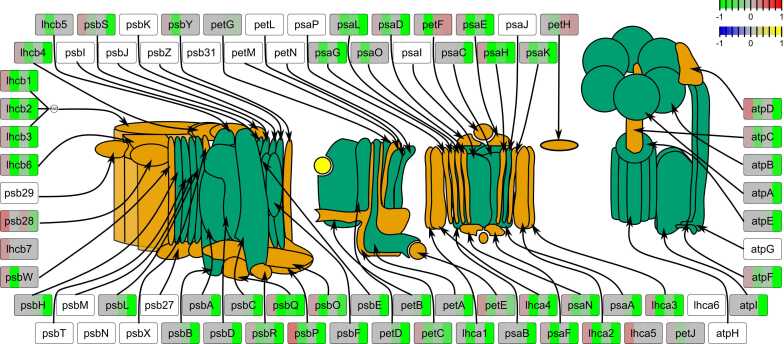
RNA and protein levels of photosystem components. Each rectangular glyph representing a photosystem component is split into 4 panels. The first two panels represent transcript levels, while the second two represent protein levels. In both the transcript and the protein section, the left panel shows the contrast of green *canal-1* leaves to wild type leaves, while right panel shows the contrast of white *canal-1* leaves to wild type leaves. Color mapping corresponds to log10-fold changes relative to wild type leaves as displayed by the color key in the top right corner. Image of photosystem components is adapted from [106] and overlaid with data mapped to SBGN glyphs.

Unfortunately, we were not able to detect the protein of interest SCO2 itself among the 7729 proteins and thus could not draw any conclusions concerning its abundance. When considering other assembly factors, we could see a slightly different pattern to that observed with the transcriptome with no significant differences at the proteome level between the two groups (Fig. 5A). Despite showing mostly lower values than green leaves, white mutant leaves did also not show any significant changes in the cumulative abundance of assembly factors (Fig. 6A). When looking at the level-scaled mean abundance of individual proteins, we could again see that orthologs of ELIP1 and DEG7 as well as orthologs of FtsH11, HCF106, PDI6/PDIL1–2#2 built a cluster of proteins with higher abundance in white leaves of *canal-1* in comparison to green leaves of the mutant and wild type plants (Fig. 5B). When looking at the individual proteins, we could see that while orthologs of ELIP1, DEG7 and FtsH11, showed some relatively high individual values only the orthologs of HCF106 and PDI6/PDIL1–2 displayed differential abundance between white leaves of the mutant and green leaves of wild type and mutant plants (Fig. 5 C). We also investigated the abundance of the proteins that finally make up the photosystem components (Fig. 5 D). The levels for white mutant leaves were extremely low and significantly lower than the protein levels in green leaves of mutant and wild type plants. Green *canal-1* leaves additionally showed a slightly but significantly reduced amount of photosystem components in comparison to green M82 wild type leaves (Fig. 5D).

### 3.4 Integrating transcriptomic and proteomic data highlights stronger downregulation of photosystem components in white *canal-1* leaves

Having both transcriptomic and proteomic data available, we set out to visualize both regarding the photosystem components on the basis of both. For this purpose we took the log10-fold changes of the contrasts comparing mutant green leaves to wild-type leaves as well as comparing mutant white leaves to wild-type leaves (Fig. 6).

At the transcript level, many of the photosystem components show a moderate upregulation in green leaves of *canal-1* leaves in comparison to wild-type leaves. White *canal-1* leaves, however, show a downregulation, when compared to green wild-type leaves. When considering the proteome, we could see that the previously detected upregulation in green leaves of the mutant in comparison to those of the wild-type, translates to normal wild-type-like protein levels. By contrast, white *canal-1* leaves displayed a reduction of photosystem component protein abundances, in comparison to wild-type leaves.

### 3.5 Metabolomics reveal amino acid accumulation in white *canal-1* leaves

**Fig. 7:**
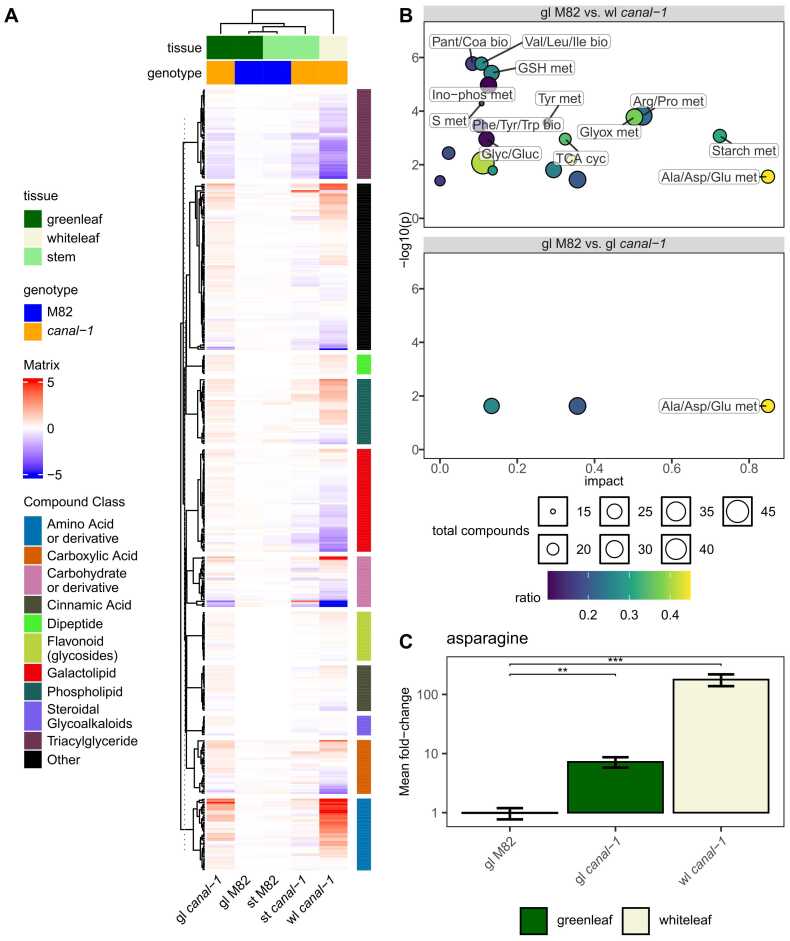
Metabolome of *canal-1* and wild type plants. A, Heatmap of all measured metabolites across stems and leaves of mutant and wild type with rows split by compound class. B, Pathway enrichment according to MetaboAnalyst. Bubbles are colored according to gene ratio and sized by their amount of total compounds in the respective pathway C, Asparagine level in *canal-1* and wild type leaves. Logarithmic scale was used for the y-axis. Statistical significance was estimated by pairwise-wilcox test for C. Significant differences are indicated by a bracket and asterisks for C (*: p ≤ 0.05; **: p ≤ 0.005; ***: p ≤ 0.0005). Full pathway names are shown in Supplementary Table S 15 and Supplementary Table S 16.

We next measured metabolites, extracted from leaves of mutant and wild-type plants (Fig. 7).

For this purpose, we used established protocols for the GC-MS-based profiling of primary metabolites[62] and the LC-MS measurement of polar secondary metabolites [63] and lipophilic metabolites [107], allowing the quantification of 126, 116 and 210 compounds, respectively (Supplementary Data S 1). We saw the strongest changes in the white leaves of *canal-1* plants (Fig. 7 A). At the bottom of the heatmap we can see a the group of amino acids and derivatives thereof, that display a strong upregulation in white leaves of *canal-1* plants under control and also drought conditions, in comparison to the respective wild-type leaves. Compounds of the classes triacylglycerides (TAGs) and galactolipids, mostly show a moderate to strong downregulation in white *canal-1* leaves. Green leaves of *canal-1* plants show similar patterns for these clusters, albeit to a much lesser extent (Fig. 7 A).

To gain a better idea of the altered pathways of primary metabolism we used Metaboanalyst to perform a KEGG pathway enrichment (Fig. 7 B). The pathway with the highest impact was “Alanine, Aspartate and Glutamate metabolism” in both comparisons (Supplementary Table S 15 and Supplementary Table S 16). We found many other pathways related to the biosynthesis and metabolism of various amino acids but also some related to carbohydrates (Fig. 7 B, Supplementary Table S 15). The compound which showed the strongest changes was asparagine. In comparison to wild-type leaves it shows roughly a ten-fold increase in green mutant leaves and a 100-fold increase in white mutant leaves (Fig. 7 C).

### 3.6 Multi-omics integration reveal shift of white mutant leaves from source to sink tissues

**Fig. 8:**
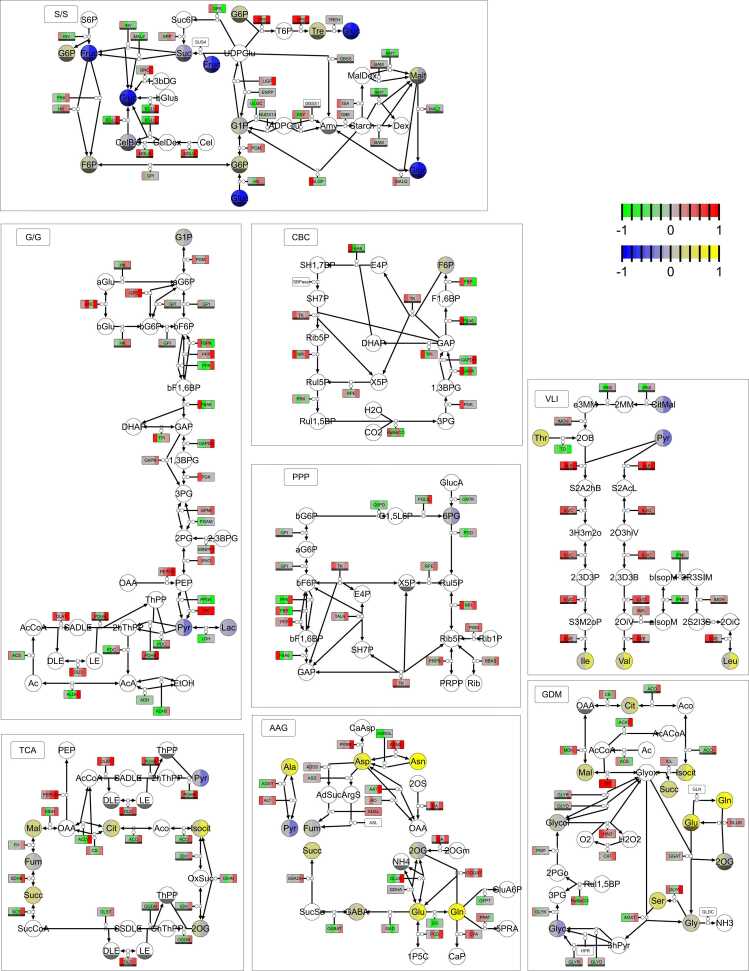
Pathway maps derived from KEGG pathways designed in SBGN format showing relative transcript, protein and metabolite levels of green and white *canal-1* leaves in comparison to wild-type leaves. Rounded rectangular glyphs are split into four sections, where the first two depict transcript levels and the last two depict protein levels. For both transcript and protein levels, the first panel displays the relative level of green mutant leaves and the second panel the relative level of white mutant leaves in comparison to wild-type leaves. Circular glyphs depict metabolites, which are split in the same way as the transcript and protein panels. Colors correspond to log10-fold changes limited from −1 to 1 as depicted by legend in upper right corner (green to red for genes and proteins, blue to yellow for metabolites). Nodes without mapping data are uncolored. Subsections correspond to separate pathways. Entities that had to be duplicated within a pathway to optimize the layout carry a black bar as a clone marker on the lower end of the glyph. Full names of genes/enzymes and compounds are found in Supplementary Data S 8. S/S: Starch and Sucrose Metabolism; G/G: Glycolysis/Gluconeogenesis; CBC: Calvin-Benson-Cycle; PPP: Pentose Phosphate Pathway; VLI: Valine, Leucine and Isoleucine Biosynthesis; TCA: TCA cycle; AAG: Alanine, Aspartate and Glutamate Metabolism; GDM: Glyoxylate and Dicarboxylate Metabolism.

Having transcriptomic, proteomic and metabolomics data at our disposal, we took the opportunity to visualize them together and investigate more deeply the pathways, revealed by KEGG pathway enrichment. Starting at the reductive part of the pentose phosphate pathway (Calvin-Benson-Cycle; CBC), we could see that green *canal-1* plants showed some upregulation of genes on a transcriptomic level, which related to a minor upregulation on the proteomic level for example for phosphoribulokinase (PRK) and RuBisCO (Fig. 8).

White *canal-1* leaves however showed a downregulation of transcripts and most proteins. Again, this is exemplified by PRK and RuBisCO, which are downregulated at both transcriptomic and proteomic levels. Several enzymes of the (oxidative) pentose phosphate pathway (PPP), some of which are shared with the Calvin-Cycle however showed an upregulation and additionally the compound 6-phospho-D-gluconate were reduced in white *canal-1* leaves (Fig. 8). In the starch and sucrose metabolism (S/S) and the glycolysis and gluconeogenesis (G/G), the compounds sucrose, glucose, fructose and maltose are strongly reduced in white *canal-1* leaves, whereas in green mutant leaves sucrose shows wild-type levels and maltose even an increase in comparison to wild type leaves. Several enzymes such as beta-glucosidase (BGLU), endoglucanase (NGLU), 6-phosphofructokinase (PFK), fructose-1,6-bisphosphatase/bisphosphate aldolase (FBPA) and fructose-1,6-bisphosphatase (FBP) show different or even opposite responses in white and green mutant leaves, suggesting that carbohydrates are catabolized in white leaves and synthesized in green leaves of the mutant (Fig. 8). Also in the TCA cycle and the glyoxylate and dicarboxylate metabolism (GDM), several catabolic enzymes are upregulated on a proteomic level in white *canal-1* leaves and downregulated in green *canal-1* leaves. In the valine, leucine and isoleucine biosynthesis pathway (VLI), the amino acids valine, leucine and isoleucine, were upregulated in green leaves but even more strongly in white leaves of the mutant. We could also see that enzymes involved in this biosynthetic pathway, namely acetolactate synthase (ALS), ketol-acid reductoisomerase (ILVC), dihydroxy-acid dehydratase (ILVD) and branched-chain amino acid aminotransferase (ILVE) displayed an upregulation, which was stronger in white leaves than in green leaves of *canal-1* plants. Similarly, in the alanine, aspartate and glutamate metabolism (AAG), the amino acids glutamate, glutamine, alanine, aspartate and asparagine were upregulated in mutant leaves. For glutamate and glutamine, we could see the upregulation of ferredoxin-dependent glutamate synthase (GLUS), NAD(P)^+^-dependent glutamate dehydrogenase (GLUD) and NADH-dependent glutamate synthase (GOGAT) which could explain the elevated amino acid levels. In aspartate and asparagine biosynthesis we found glutamine-hydrolysing asparagine synthase (ASNS) and in white leaves also aspartate aminotransferase (AAT) to be upregulated (Fig. 8). These observations thus supported the results from the KEGG pathway enrichment.

### 3.7 Correlation network construction supports a hub function of SCO2

**Fig. 9:**
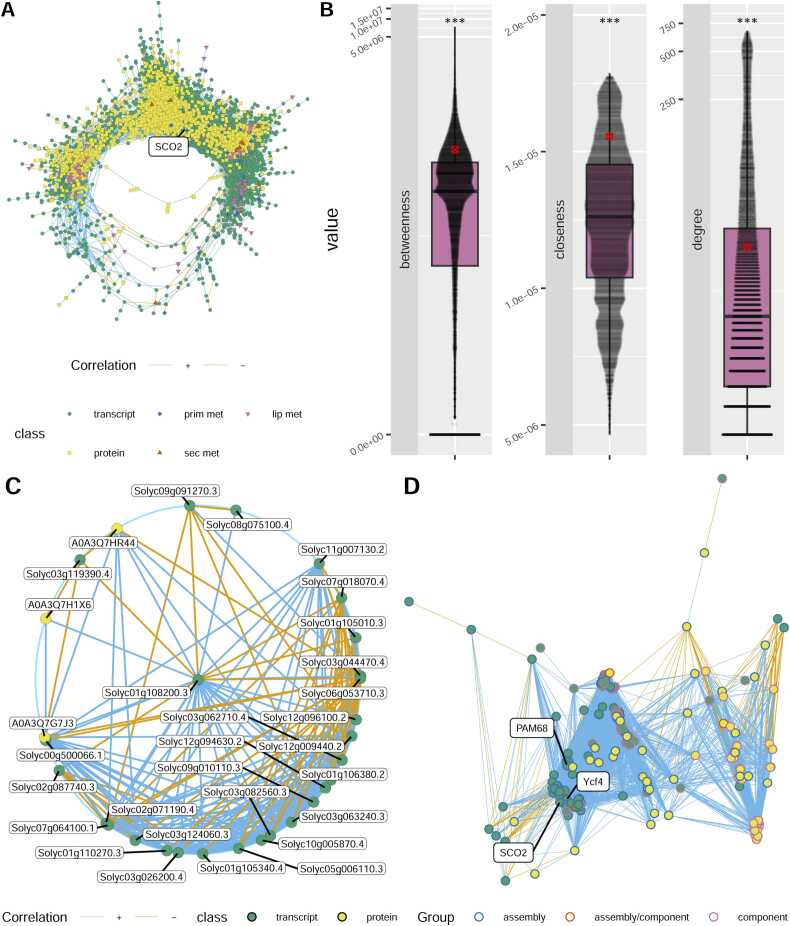
Correlation network graph, subnetworks and network metrics. A, Single connected correlation network graph based on spearman`s correlation value between levels of transcripts, proteins, primary metabolites, secondary metabolites and lipophilic compounds across leaf and stem wild-type and mutant samples. Vertices correspond to individual transcripts, proteins or compounds and are displayed by filled points, colored according to their vertex class. Vertices are connected through edges colored by the sign of the correlation. Graph layout was generated by a progressive multidimensional-scaling algorithm, using the inverse of the edge-weights. Correlation-value threshold = 0.95, n_vertex_ = 12478; n_edge_ = 326539. B, Betweenness, closeness and degree of SCO2 transcript in relation to the overall network. All boxplots show the interquartile range (IQR) between the first and the third quartile as the box, with the median indicated by a black center line. Whiskers extend from quartiles to most extreme points with a maximum of 1.5 x IQR. Points beyond that range are considered outliers but overlaid by individual data points, shown as quasirandom. The respective SCO2 value is indicated by a red cross-circle. Y-axis is transformed by log(1 +x) to accommodate 0 values as well as large outliers. C. Correlation network graph of direct SCO2 neighbors at correlation value threshold 0.95. D, Correlation network graph of photosystem assembly factor genes and proteins at correlation value threshold of 0.75. In C + D, vertices correspond to individual transcripts or proteins and are displayed by filled points, colored according to their vertex class. Vertices are connected through edges colored by the sign of the correlation. In D the outline of the points (stroke) is colored according to the functional grouping. Graph layout was generated by a circular layout algorithm for C and a progressive multidimensional-scaling algorithm for D, using the inverse of the edge-weights. Statistical significance was estimated by single-wilcox test for B. Significant differences are indicated by asterisks for B (*: p ≤ 0.05; **: p ≤ 0.005; ***: p ≤ 0.0005).

The availability of this multi-omics dataset for the 15 samples from mutant and wild type stems and leaves also gave us the opportunity to explore how transcript-, protein- and metabolite levels correlate to one another. We therefore calculated the Spearman-correlation value for all pairwise-combinations, resulting in over 900 million correlation values. In order to reduce the number of correlations to a manageable amount we directly filtered out correlation values below 0.75, which still left us with more than 29 million correlations. For an overview, we applied an even more stringent threshold of 0.95, created a correlation network graph using absolute correlation values as edge-weights and only retained the largest connected graph for a visualization (Fig. 9 A). This resulted in a graph with 12478 vertices and 326539 edges. (Fig. 9 A).

Due to its function in canalization of yield, we wondered, whether SCO2 shows typical properties of a network hub and therefore compared different network metrics of SCO2 to those of the overall network (Fig. 9 B).

When comparing the degree, closeness and betweenness of SCO2 to the rest of the filtered network, we realized that the values of SCO2 were indeed significantly higher than the (pseudo-)median of the overall network (Fig. 9 B, Supplementary Table S 17). While the degree of 29 of SCO2 is only somewhat higher than the median degree of the network, the metrics closeness and betweenness both exceed the third quartile of the overall network (Fig. 9 B). Focusing further on our gene of interest we queried the direct neighbors that SCO2 has at the 0.95 threshold (Fig. 9 C). As already observed by the degree of SCO2, we could now see the 29 neighbors which strongly correlated to SCO2 and also among themselves (Fig. 9 C, Table 2).

**Table 2:** Direct neighbors of SCO2 transcript with absolute correlation-value above 0.95.

Gene	ITAG4.0 annotation	Correlation value
Solyc06g053710.3	ethylene receptor homolog (ETR4)	−0.971428571
Solyc09g091270.3	cotton fiber protein	−0.964285714
Solyc01g105010.3	Non-specific lipid-transfer protein-like protein	−0.960714286
Solyc02g087740.3	Plant cysteine oxidase 2	−0.957142857
Solyc07g018070.4	Double Clp-N motif-containing P-loop nucleoside triphosphate hydrolases superfamily protein	−0.957142857
Solyc03g119390.4	bHLH transcription factor 026	−0.95
Solyc01g106380.2	hypothetical protein	0.95
Solyc01g110270.3	RNA-binding CRS1 / YhbY (CRM) domain protein	0.95
Solyc03g082560.3	Aldo-keto reductase/ oxidoreductase	0.95
Solyc12g094630.2	Low-density receptor-like protein	0.95
Solyc01g105340.4	Chaperone protein DnaJ	0.953571429
Solyc02g071190.4	Carboxyl-terminal-processing protease-like protein	0.953571429
Solyc03g026200.4	protein COFACTOR ASSEMBLY OF COMPLEX C SUBUNIT B CCB2, chloroplastic	0.953571429
Solyc03g044470.4	red chlorophyll catabolite reductase	0.953571429
Solyc03g062710.4	protein NDH-DEPENDENT CYCLIC ELECTRON FLOW 5	0.953571429
Solyc12g096100.2	Protein PAM68, chloroplastic	0.953571429
A0A3Q7HR44	NA	0.953571429
Solyc00g500066.1	Photosystem I assembly protein Ycf4	0.957142857
Solyc03g063240.3	Pyridoxamine 5'-phosphate oxidase-related FMN-binding protein	0.957142857
Solyc03g124060.3	Polyketide cyclase/dehydrase and lipid transport superfamily protein	0.957142857
Solyc08g075100.4	Initiation factor 4 F subunit (DUF1350)	0.957142857
Solyc11g007130.2	Major facilitator superfamily	0.957142857
A0A3Q7H1X6	NA	0.967432647
Solyc07g064100.1	Chlororespiratory reduction 41	0.967857143
Solyc12g009440.2	DnaJ/Hsp40 cysteine-rich domain superfamily protein	0.967857143
A0A3Q7G7J3	NA	0.967857143
Solyc05g006110.3	Serine/Threonine-kinase	0.971428571
Solyc10g005870.4	Peptidylprolyl isomerase	0.971428571
Solyc09g010110.3	Chaperone protein dnaJ-related protein	0.975

The first observation we could make is that most of the SCO2-neighbors are transcript nodes, with only 3 protein nodes. Out of the correlations to SCO2 only 6 were negative, while 23 were positive. The strongest negative correlation was to the transcript Solyc06g053710.3, which is annotated as ethylene receptor homolog (ETR4) (Fig. 9 C, Table 2). The strongest positive correlation was to the transcript Solyc09g010110.3 which is annotated as Chaperone protein dnaJ-related protein. Besides this gene we also found two more transcripts carrying an annotation relating to DnaJ proteins, namely Solyc12g009440.2 and Solyc01g105340.4. Solyc09g010110.3 is orthologous to the ORANGE gene in Arabidopsis (AT5G06130.2), which has been shown to be involved in carotenoid biosynthesis by binding to phytoene synthase [108]. Other interesting correlation partners were Solyc12g096100.2 and Solyc00g500066.1, which carry the annotations Protein PAM68, chloroplastic and Photosystem I assembly protein Ycf4, respectively. These are also orthologous to the Arabidopsis assembly factors PAM68 (AT4G19100) and Ycf4 (ATCG00520), respectively, which we found in our orthology search before (Supplementary Table S 5). This observation made us wonder whether SCO2 transcript abundance also correlates to the abundance of other photosystem assembly factor transcripts or proteins, albeit with a lower correlation value and how strong the correlation is to the components that the photosystem finally consists of. We therefore reinvestigated the whole dataset with the more relaxed threshold of 0.75 and looked at correlations between photosystem components and assembly factor transcripts and proteins. This resulted in a dense network of transcript and protein nodes connected mostly through positive correlations (Fig. 9 D). Both transcripts and protein nodes connected more strongly to members of their own class than to members of other classes. We found that SCO2 clustered together mostly with transcripts of other assembly factors such as Ycf4 and PAM68 towards the left of the network. Towards the middle we found a cluster with mostly transcript nodes of assembly factors and towards the right half of the network we found more protein nodes, with a densely connected cluster of protein nodes of photosystem components (Fig. 9 D). Using a hierarchical clustering algorithm of all raw unfiltered correlation values of the previously discussed transcripts and proteins and plotting them on a heatmap (Supplementary Figure S 11), resulted in a similar observation.

### 3.8 Photosynthesis in green leaves of *canal-1* is increased to compensate impaired photosynthesis in white leaves

**Fig. 10:**
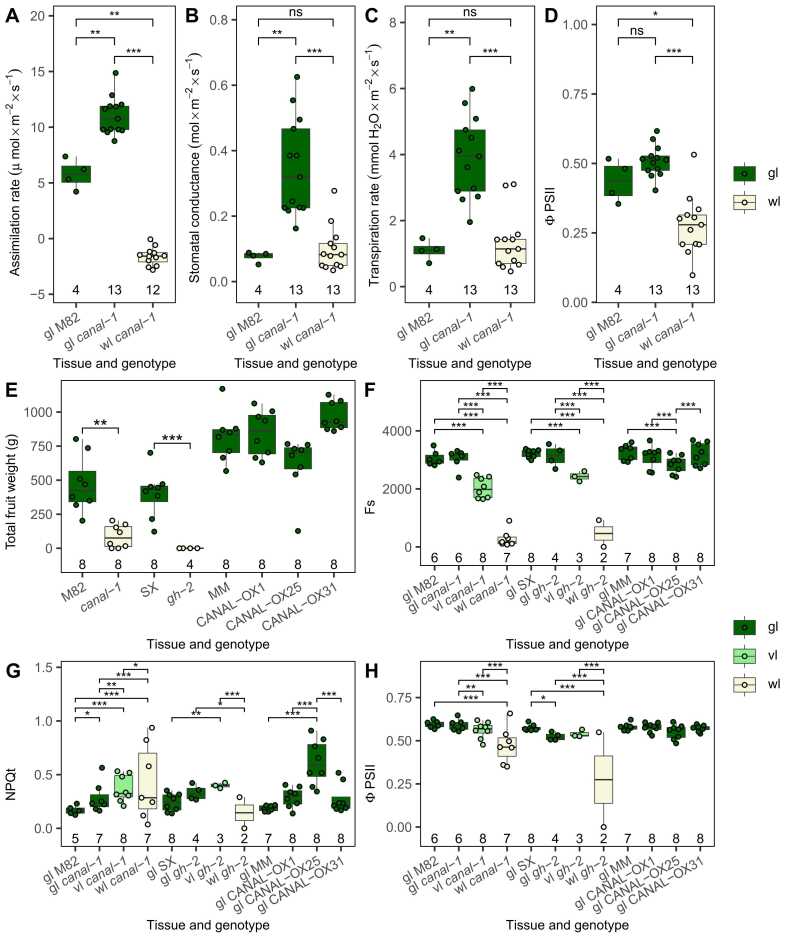
Photosynthetic measurements as conducted by LiCOR in the light (A-D) and yield and photosynthetic parameters on wild type, mutants and overexpression lines as conducted by multispeq device (E-H). A, Photosynthetic assimilation rate. B, Stomatal conductance. C, Transpiration rate. D, Quantum yield of photosystem II (ϕPSII). E, Total fruit weight (g) of different lines. F, Baseline Fluorescence (Fs). G, Non-photochemical quenching (NPQt). H, Quantum yield of photosystem II (ϕPSII). Boxes and points are filled according to tissue (gl = green leaves: green, vl = variegated leaves: bright green, wl = white leaves: beige). Statistical significance was estimated by pairwise-wilcox test for A-D and pairwise-t-test for E-H. Significant differences are indicated by a bracket and asterisks (*: p ≤ 0.05; **: p ≤ 0.005; ***: p ≤ 0.0005).

We were additionally interested to see, whether the changes observed via the –omics analysis were also reflected in the photosynthetic activity and therefore estimated several photosynthetic parameters using a LiCOR (Fig. 10 A-D).

The assimilation rate of green leaves of the *canal-1* plant was significantly increased in comparison to wild-type leaves, whereas white mutant leaves showed a negative assimilation rate, which means, that they were actually carrying out net respiration (Fig. 10 A). Additionally, green leaves of the *canal-1* mutant had a significantly increased stomatal conductance and transpiration rate compared to wild-type leaves, whereas white mutant leaves showed wild-type levels (Fig. 10 B and C). Regarding the quantum yield of photosystem II, green leaves of mutant and wild-type showed similar levels, whereas white *canal-1* showed a statistically significant lower level (Fig. 10 D).

In a final experiment, we grew the *canal-1* mutant, the allelic *ghost-2* mutant, and three over-expressing lines, together with their respective wild-type cultivars, M82, Sioux (SX) and MoneyMaker (MM) in a greenhouse experiment (Fig. 10 E-H).

As observed before, the *canal-1* mutant displayed a significantly decreased yield, as did the *ghost-2* mutant (Fig. 10 E). In fact, the effect of the *ghost-2* mutant, was so strong, that neither of the plants was able to produce any fruit (Fig. 10 E). Lines overexpressing SCO2 did not show significant differences regarding fruit weight in relation to the MoneyMaker wild-type (Fig. 10 E). However, Line CANAL-OX31 displayed a significantly earlier flowering time and an increased number of fruits per plant (Supplementary Figure S 12), traits that could be of high agronomic interest.

Due to the increased sample size, we did not use the LiCor for photosynthetic measurements in this experiment but rather used a small multispeq handheld device. On the other hand, as an intermediate between green leaves and white leaves, we now also included variegated leaves, i.e. leaves that had both white as well as green sectors, in the measurement. The steady-state fluorescence measurements revealed significantly reduced levels for variegated mutant leaves and an even greater reduction for white leaves, when compared to the green leaves of the corresponding wild-type (Fig. 10 F). Green mutant leaves on the other hand showed wild-type fluorescence levels. Interestingly, green leaves from the CANAL-OX25 line also showed a significant reduction in Fs, when compared to MoneyMaker leaves. More or less the opposite effect could be observed for non-photochemical quenching (NPQt) (Fig. 10 G). Here, variegated mutant leaves showed an increase in NPQt. White leaves of *canal-1* also showed an NPQt increase, whereas white leaves of *ghost-2* showed a reduction. Finally, the quantum yield of photosystem II was similar to that observed with the LiCor (Fig. 10 H), while overexpressor lines were invariant from wild-type plants.

## 4 Discussion

In the current study, we presented the molecular characterization of the variegated *canal-1* mutant in tomato. Leaves showed different patterns and degrees of variegation, from almost completely green over variegated to completely white, with white leaves being devoid of photosynthetic pigments, whereas green mutant leaves contained wild-type levels. Transcriptomic analysis revealed the enrichment of photosynthesis-related terms and highlighted upregulation of photosystem assembly and photosystem component genes in green mutant leaves, while white mutant leaves showed wild type levels of photosystem assembly genes, with the exception of orthologs of ELIP and DEG genes, which showed high outlying values. These changes seen at the transcriptomic level, were paralleled at the proteome level, with the proteome of green *canal-1* leaves being largely invariant from wild-type leaves, but white *canal-1* leaves displayed strong deficiencies in proteins related to photosynthesis. Here we did not only see changes in the thylakoid membrane proteins, but also in RuBisCO, which was downregulated in white mutant leaves. More generally we do see some differences between proteome and transcriptome. While a certain discrepancy could be expected and has been shown before [109], [110], some additional variation may result from the fact that proteomics more easily pick up the products of plastid-encoded genes, than the transcriptomic analysis. The metabolome showed a strong upregulation of amino acids and a downregulation of TAGs and galactolipids in white *canal-1* leaves. The results of our study report similar findings to the earlier more focused studies in *Arabidopsis thaliana* and *Lotus japonica*. Indeed, the tomato *canal-1* mutant more similar resembles the *sco2* mutants of Lotus in that their true leaves are affected and display a variegated phenotype under long-day conditions and similarly the levels of PsaB, PsaL, PsbC and PsbD are also decreased in white mutant leaves as was shown for Lotus. By contrast to the results described in Lotus, PsbO and PsbQ are not more abundant at the protein level in tomato. In fact, PsbP - which is also part of the oxygen-evolving complex- was the only differentially abundant protein in green *canal-1* leaves. On the other hand, all three of these proteins (PsbO, PsbP and PsbQ) show transcriptional upregulation in green leaves but downregulation in white leaves (Fig. 6). This is underlined by the fact that PSII oxygen evolving center was one of the most significantly upregulated terms in green mutant leaves compared to wild type but also compared to white mutant leaves. Considering that results from green leaves might be less disturbed by pleiotropic effects, and thus more accurately show the direct effect of the mutation, these results could suggest that SCO2 is particularly relevant in aiding the (re-)assembly of PsbP into OEC-less PSII precursors during regular assembly or repair of PSII [111]. Also similar to previous results is the reduction of lhcb1 in white *canal-1* leaves, which has been shown on a transcriptomic level in Arabidopsis [29] and on a proteomic level in Lotus [27]. A reduction of D1 and LhcB protein had also been shown for Arabidopsis by an earlier study, which however found little changes in gene expression [24].

Given that correlation analysis clustered the SCO2 transcript closer to assembly genes than to other components of the photosystems, we doubt that SCO2 directly affects transcription or translation of photosystem components and feel that they are more likely to be secondary consequences. Rather, as we discuss in detail below, we feel that SCO2s primary role is in photosystem assembly and that the chloroplast biogenesis may be affected due to the function as assembly component. That said, correlation analysis revealed above median levels of different network metrics (degree, closeness and betweenness), for the SCO2 transcript and high correlation values to a range of other putative photosystem assembly genes. Furthermore, pathway integration highlighted the increased carbohydrate catabolism in white leaves and confirmed the upregulation of amino acid biosynthetic pathways. Moreover, photosynthetic measurements showed a reduction of activity in variegated and white leaves of *canal-1*, whereas green mutant leaves showed an upregulation of some parameters. Thus, whilst falling short of defining the exact underlying causal mechanisms, we want to highlight two characteristics of our work that distinguish it from the existing literature. First, we have quantitative data for the whole transcriptome and are able to observe changes in gene expression on a much broader range and with higher precision than this has been done previously [24], [29] and secondly, we separately analyzed white and green leaves/leaf sectors and could contrast them against each other. This of course was not possible for Arabidopsis but was also not performed for Lotus [27]. We discuss below the collective results both with respect to the function of SCO2 and with regard to its previous identification as a canalization gene [23].

Due to the changes observed in the multi-omics analysis, we suggest that an adequate amount of SCO2 activity is indispensable for appropriate thylakoid development. The mutation of the *canal-1* mutant is in close proximity to a conserved zinc finger binding domain that is crucial for catalytic activity [23], [30]. Unfortunately, due to the absence of an available antibody we were not able to detect the levels of the SCO2 protein. This is also underlined by the phenotype of other mutants described in literature. Two CRISPR mutants have previously been described [23]. The strongest phenotype is displayed by a mutant with an early stop codon before the conserved zinc finger domain, which produces a truncated protein. It has pale cotyledons and fails to build true leaves. Slightly less severe was the phenotype of another CRISPR/Cas9 mutant, with a frame shift that resulted in a change of amino acids 15 to 43. This mutant also developed variegated leaves, but did not survive past the first true leaves. The *ghost-2* mutant, similarly to *canal-1*, is an EMS mutant with a single point mutation, leading to the change of an amino acid within the conserved zinc finger domain. Despite also showing a variegated leaf phenotype, this mutant has been described to not show the unstable variegation phenotype and produce uniformly large plants [23]. This is, however, in disagreement with our observation that plants harboring this mutation show a more severe phenotype and are characterized by being unable to produce any fruit. Additionally, despite the increased variation in fruit yield, *canal-1* mutant plants did not vary as strongly in the greenhouse as was observed in the field. Therefore, the discrepancies between our results and the field trials may be attributed to the effect the different environments had.

The RNAseq experiment did not yield any significant differences in gene expression in the causal gene SCO2 between mutant and wild type leaves suggesting that the protein was expressed and likely transcribed but non-functional. Moreover, despite being able to detect 7729 proteins, which is similar to the number reported in previous experiments or even higher [112], [113], [114], we were not able to detect SCO2 among them and cannot say whether protein levels were changed. This was most likely just the technical limitation of the proteomics in achieving complete coverage of the whole proteome. More generally however, the upregulation of gene expression, specifically of photosystem assembly components, in green mutant leaves in comparison to wild type leaves, suggests an overcompensation in response to an, at least partially, impaired protein function in SCO2. The correlation network analysis confirmed the high correlation of this expression pattern between the SCO2 transcript and other photosystem assembly components. Our overcompensation hypothesis is further supported by the wild-type-like protein levels in these leaves. Indeed, white *canal-1* leaves showed wild-type transcript levels of most photosystem assembly components. Exceptions to this statement were the upregulation of genes orthologous to ELIP and DEG genes. In de- etiolating seedlings ELIPs were demonstrated to be responsible for the etioplast to chloroplast transition [115], however since the true leaves develop in the light, ELIP accumulation may be rather indicative of high photooxidative stress in white leaf sectors [116]. This could lead white leaf sectors to get trapped in a stage similar to pre-de-etiolation, wherein thylakoid development is arrested and misfolded proteins get degraded by DEG protease. As a consequence of such a scenario proper thylakoids would be unable to develop and protein levels of photosystem components would be significantly reduced. Being unable to photosynthesize, white mutant leaves rather behave as a sink tissue on a metabolic level. This can be seen by the depletion of several mono- and disaccharides like sucrose, maltose, glucose and fructose and the upregulation of enzymes involved in their catabolism in the glycolysis, like PFK and GAPDH. On the other hand, enzymes along the same pathway in the anabolic direction, like GAPA and FBPA are downregulated. By contrast, green mutant leaves seem to be able to compensate for the lack of photosynthates by an upregulation of photosynthesis and an altered carbon allocation. This can also be seen for example in the enzymes GAPA and FBPA showing the opposite effect than white leaves towards an increased sugar anabolism. The upregulation of amino acids is likely a secondary effect, as a general response to stress, which is supported by transcriptomic and proteomic changes in enzymes involved in the biosynthesis of amino acids, like ASNS, GOGAT, ILVC, ILVD and ILVE. Downregulation of TAGs and galactolipids may be related to the missing thylakoid membranes. The increased photosynthesis in green mutant leaves is displayed by a higher assimilation rate. This in turn, however, requires green leaves to open their stomata wider, which consequently comes at the cost of increased transpiration.

The compensation response described above fits to a threshold model described for other variegation mutants [117], [118]. This model suggests a base-level of either protein function or abundance, to be necessary for the development of functioning photosystems. Furthermore, it is well known that the stoichiometry of proteins is important for thylakoid development [27]. In this regard, SCO2 has previously been described as a “scale” that balances different photosystem components [27]. All of this applied to the observations in the *canal-1* mutant, could mean that there is a certain critical level of SCO2 function, which if surpassed enables the formation of thylakoids and consecutively the development of green leaf sectors. If this level is however undershot, thylakoid formation is not achieved and leaf sectors stagnate in an undeveloped state. This may be exacerbated by the fact that at least photosystem II is especially vulnerable to photodamage in early development [119]. It seems reasonable to suggest that the larger the total area of photosynthetically active tissue, the larger the fruit mass it can sustain. Each wild type plant will usually have a relatively uniformly green canopy, thus the percentage of photosynthetically active tissue can be considered to be close to 100 %. As described earlier, the variegation in *canal-1* mutant plants leads to a large variation of green and white tissue area within a single leaf and plant and thus also a variation from plant to plant. A recent study, describing an FtsH mutant with a similar phenotype to the one described in our study, has nicely quantified the shades of green in separate variegated mutant leaves and demonstrated exactly this variance from leaf to leaf [120]. While we have not quantified this variance ourselves in the *canal-1* mutant an increased variation seems plausible based on the fluorescence measurement we performed on white, green and randomly selected variegated leaves. From here it seems obvious that a plant-to-plant variation of total photosynthetically active tissue, may lead to a variation in yield, which may be further aggravated according to our hypothesis that white leaves act as additional sink tissues, thus linking the variation of photosynthetic tissue to the variation in yield. This theory could also explain why our results from greenhouse experiments were different from those obtained in the field. Light intensity is usually much higher in the field in comparison to a greenhouse. For young mutant plants, with an impaired photosystem assembly protein, this may present a stress factor, which could early on lead to the development of white sectors. Additionally, a necessary increase in stomatal conductance in mutant green leaves, likely makes mutant plants more vulnerable to heat and drought stress. This may be an additional fact that explains the large variance of small and large plants in the field.

As we are interested in SCO2 as a canalization gene, we felt this data should put it into the context of known canalization genes. It has been suggested that such genes are so-called hub genes [121]. The most well-described canalization gene, in this respect, is the chaperone heat shock factor 90 (*HSP90*)[122]. Another hub gene, which was found to affect the variation of glucosinolate levels, circadian periodicity as well as flowering time, is the gene *EARLY FLOWERING 3* (*ELF3*), which is known to be generally involved in the circadian pathway [123], [124]. Similarly, the *ERECTA* gene, which is a known pleiotropic developmental regulator, was suggested to influence canalization of rosette leaf number [18], [125]. Based on these examples, we could say that there are some hub genes, which are more directly involved in specific gene networks as well as those, like HSP90, that are only peripherally connected but affect many processes [122].

In congruence with these examples, we can see several overlaps to SCO2. Firstly, on the molecular level SCO2 is also a DnaJ-like chaperone that is suggested to interact with other proteins [25], [29]. The demonstrated catalytic activity of SCO2 as a disulfide isomerase further suggests it might accelerate the folding of photosystem subunits, which contain cysteine residues [30]. Even though the specific function of SCO2 in thylakoid assembly, would qualify it as a gene of a specific gene network, we know that it also has a global effect on yield [23]. We would also argue that SCO2 is involved in a larger gene network, which extends beyond its immediate interaction partners. Our correlation analysis has shown that SCO2 has 29 direct neighbors, which makes its node degree already significantly higher than the median node degree of the correlation network, again highlighting the role of SCO2 as a hub gene. Beyond that SCO2 also displayed a higher betweenness than the median of the network, suggesting its involvement in a larger network. Our results also revealed that SCO2 expression correlates more closely with the expression of other putative assembly factors and only more distantly to photosystem components. SCO2 transcript showed the highest positive correlation to Solyc09g010110, an ortholog to the ORANGE gene in Arabidopsis, which has been shown to play a key role in chromoplast differentiation by posttranscriptionally regulating phytoene synthase [108]. Similarly, Solyc09g010110 in tomato (also called OR-like) has also been suggested to play an important role in tomato fruit ripening and carotenoid accumulation [126]. Other interesting genes that showed a high correlation to SCO2 transcripts were orthologs of Ycf4 and PAM68, which are known factors in the assembly of photosystem I and II respectively [45], [127]. Ycf4 binds to different PSI subunits, but in tobacco seems to be non-essential for photosystem I assembly [128]. PAM68 has been shown to be involved in early PSII assembly, interacting both with early assembly intermediates as well as other assembly factors [129]. Similarly, SCO2 may fulfill analogous roles to ensure the proper folding of photosystem proteins during photosystem assembly or repair. The proper establishment of photosystem components, in which SCO2 likely plays an important role, could be described as a multi-layered regulation mechanism as detailed below.

The proper assembly of photosystem components requires a tight coordination between nucleus and plastid on multiple levels [130]. On a first level, during chloroplast biogenesis, there is a two-phase transcriptional regulation, with anterograde and retrograde signaling between nuclear and plastid genome [131]. Through a second layer of control, the translation of some photosystem subunits is autoregulated by the assembly state, to ensure the correct stoichiometry [132]. As mentioned above SCO2 has been assigned a similar role balancing the correct ratio of PSI/PSII complexes and LHC proteins in *Arabidopsis thaliana* and *Lotus japonicus* [27] and our results suggest a similar mechanism in tomato. As this step concerns readily translated proteins, this could be regarded as a third layer to ensure appropriate stoichiometry, where SCO2 plays a key role. It therefore seems plausible that reduced SCO2 function could decanalize the development of proper photosynthetic tissue and as a consequence increase variation of plant yield.

## 5 Conclusion

Although we are largely satisfied with our explanation of how SCO2 affects canalization of plant yield, further, more targeted experiments with higher spatial and temporal resolution would likely be required to gain a more precise understanding of the exact mechanistic role of the SCO2 protein. Nevertheless, our work underlines the importance of the SCO2 protein in thylakoid formation and explains the differences between white and green sectors of the *canal-1* mutant, by different responses on a transcriptome, proteome and metabolome level as well as physiological response. It also largely explains how SCO2 can stabilize both metabolism and morphology. It is our contention that our work alongsided the earlier work on Hsp90 suggests that other chaperone-like proteins may be interesting candidate genes as a means to gain a fuller understanding of how this important and highly agriculturally relevant phenomenon is achieved. Overexpression of SCO2 did not result in increased yield under greenhouse conditions but may be tested in future studies in relation to field conditions or simulated stress. Whether this is transferable and indeed if it is useful for breeding of superior agronomic traits awaits future research.

## CRediT authorship contribution statement

**Josef Fisher:** Resources. **Micha Wijesingha Ahchige:** Writing – review & editing, Writing – original draft, Visualization, Formal analysis, Data curation. **Alisdair Fernie:** Writing – review & editing, Supervision, Project administration, Funding acquisition, Conceptualization. **Dani Zamir:** Supervision, Resources. **Saleh Alseekh:** Writing – review & editing, Supervision, Project administration, Funding acquisition, Conceptualization. **Nicola Illing:** Supervision. **Aleksandra Skirycz:** Supervision. **Ewelina Sokolowska:** Methodology, Formal analysis, Data curation. **Rafe Lyall:** Software, Methodology, Formal analysis.

## Declaration of generative AI and AI-assisted technologies in the writing process

During the preparation of this work the authors used Grammarly (©Grammarly Inc., CA, USA) in order to check grammar, interpunctation and improve readability of the text. After using this tool, the authors reviewed and edited the content as needed and take full responsibility for the content of the publication.

## Declaration of Competing Interest

The authors declare no conflict of interest.

## Data Availability

Raw data, scripts and metadata, necessary to reproduce the results are publicly available in an annotated research context (ARC), hosted on the DataHUB of the DataPLANT consortium for FAIR data management [1] under https://git.nfdi4plants.org/projects/487 and published under a CC-BY 4.0 license (https://doi.org/10.60534/ecjy4-re035). The tools SWATE, ARCCommander and ARCitect were used to create and manage the ARC.

## Appendix A Supplementary material

.

Supplementary Data S 1: List of metabolic features measured across all platforms. Supplementary Data S 2: Metabolite levels of all individual samples relative to respective wild type samples. Supplementary Data S 3: Expression matrix of raw sequencing reads as produced by LSTrAP. Supplementary Data S 4: Expression matrix of deseq2-normalized reads and log2-fold changes and p-values of respective contrasts. Supplementary Data S 5: Correspondence table between Solgenomics and Entrez Gene IDs. Supplementary Data S 6: Table of protein Groups as produced by MaxQuant software. Supplementary Data S 7: Table of quantile-normalized LFQ values of protein abundance and log2-fold changes and p-values of respective contrasts. Supplementary Data S 8: EC number, enzyme name, symbol, KO and corresponding KEGG maps used in SGBNview figures. Supplementary Data S 9: KEGG Compound ID, compound name and symbol used in SBGNview figures.
